# Genetics of Early-Onset Alzheimer Dementia

**DOI:** 10.1100/tsw.2003.39

**Published:** 2003-06-16

**Authors:** Rosa Rademakers, Marc Cruts, Christine Van Broeckhoven

**Affiliations:** Department of Molecular Genetics, Flanders Interuniversity Institute for Biotechnology (VIB), University of Antwerp, Antwerpen, Belgium

**Keywords:** Alzheimer dementia, neurodegeneration, mutations, linkage analysis, association analysis, mouse models

## Abstract

Alzheimer's dementia (AD) is the most common degenerative disorder of the central nervous system. Although the onset of dementia is above 65 years of age in the majority of the patients (late-onset AD, LOAD), a small subgroup of patients develops AD before 65 years of age (early-onset AD, EOAD). To date 3 genes responsible for EOAD have been identified: the amyloid precursor protein gene (APP), presenilin 1 (PSEN1) and presenilin 2 (PSEN2). PSEN1 is the most frequently mutated EOAD gene with a mutation frequency of 18 to 50% in autosomal dominant EOAD. In addition, the e4 allele of the gene encoding apolipoprotein E (APOE) was identified as a risk factor for both LOAD and EOAD. Many studies reported other susceptibility genes, but the APOE?4 alelle has been the only risk factor that was consistently replicated in all AD populations. Extensive cell biology research in the past ten years led to the hypothesis that the 4 EOAD genes lead to AD through a common biological pathway resulting in abnormal APP processing by subtle different mechanisms. Now, transgenic mice are produced to study the influence of EOAD mutations in vivo, eventually leading to the development of novel therapeutic strategies.

### INTRODUCTION

Alzheimer's disease (AD) is a degenerative disorder of the central nervous system that causes progressive memory loss and cognitive decline during mid to late adult life, leading to dementia and ultimately death. AD is the most common form of dementia in the elderly, and represents the fourth largest cause of death in the developed world. In the near future, the impact of AD on our society will dramatically increase since, as populations live longer, the number of elderly people continues to grow.

The clinical symptoms of AD display a substantial overlap with clinical symptoms observed in other neurodegenerative disorders with dementia as accompanying feature, such as frontotemporal dementia (FTD) and Creutzfeldt Jacob's disease (CJD). Therefore a definite diagnosis of AD can only be obtained at autopsy. Extensive neuronal loss, and two particular brain lesions in the cerebral cortex, hippocampus, and amygdala characterize AD: the senile plaques (SPs) and neurofibrillary tangles (NFTs). The SPs are compact extracellular deposits that consist of a large amyloid β (Aβ) core surrounded by dystrophic neurites. NFTs are intraneuronal inclusions of paired helical filaments (PHF) that are mainly composed of hyperphosphorylated microtubule associated protein tau (MAPT).

Based on the age at which the first clinical symptoms become apparent, AD is divided into two subgroups. By convention, an onset age of 65 years is used as the cut-off value to distinguish between early-onset or presenile AD (EOAD) and late-onset or senile AD (LOAD). EOAD patients are clinically and pathologically identical to LOAD patients, although the disease progression is more rapid and the brain pathology more pronounced in EOAD patients.

This e-review on the genetics of EOAD gives an overview of the four known genes involved in the etiology of EOAD and describes a selection of candidate genes reported to increase the susceptibility to EOAD. The structure of these genes and the influence of mutations on the processing of these genes, both *in vitro* and *in vivo*, will be discussed. Moreover, the nature of this review allowed us to provide a direct link between the schematic representation of the EOAD genes in this article and the information included in the AD mutation database (Marc Cruts, http://molgen-www.uia.ac.be/ADMutations/)[1].

### MOLECULAR GENETICS OF EOAD

The two most important risk factors for AD are age and a positive family history of AD[2]. Population studies have shown that less than 1% of 60–64 year olds are affected in contrast to 40% of those older than 85 years[3]. Although this implicates that AD mostly occurs in the elderly, 1–2% of all affecteds are EOAD patients. In the majority of these EOAD patients the disease aggregates within families, and in 10% of these families the inheritance pattern is consistent with an autosomal dominant disorder with an age-dependant penetrance (<1% of total AD patients).

The rare autosomal dominant presenile dementia families have been crucial for the identification of AD genes. Since the underlying biochemical defect in AD is unknown, the positional cloning strategy was used to identify genes involved in the AD pathogenesis. Positional cloning is based on co-segregation analysis of a disease with polymorphic genetic markers from known chromosomal loci, without any knowledge of the function or localization of the disease gene prior to the analysis. The term linkage is used when a specific chromosomal fragment is more frequently shared among affected individuals than would be expected to occur by chance. The degree of linkage is expressed as a logarithmic "log of the odd" LOD-score. A LOD-score of 3, meaning that chances for linkage are 1000 to 1, is generally accepted as conclusive for linkage. Because the interpretation of genetic studies, especially for complex traits, has previously led to misinterpretations, Kruglyak and Lander defined the terms "suggestive" and "significant" linkage. If these definitions are applied to, for example, the identification of a disease locus in a genome wide scan, suggestive linkage corresponds to a LOD-score of 2.2 whereas a LOD-score of 3.6 would be sufficient for significant linkage[4].

Using the positional cloning strategy, three genes responsible for EOAD have been identified: the amyloid precursor protein (APP) gene located on chromosome 21 and the two homologous presenilin genes, presenilin 1 (PSEN1) and presenilin 2 (PSEN2), located on chromosomes 14 and 1, respectively. In addition, a variation in the gene encoding apolipoprotein E (APOE) on chromosome 19 was identified as a risk factor for both LOAD and EOAD in familial and sporadic AD patients.

### APP

#### Identification of APP: Gene and Protein Structure

Several events led to the discovery of mutations responsible for AD in the APP gene. First it was recognized that all aged Down syndrome patients with a complete trisomy 21 developed an AD-like brain pathology[5,6]. As a consequence, linkage analysis with markers on chromosome 21 was performed in LOAD and EOAD families and suggestive linkage to 21q11.2-21q21 was found[7,8]. Although the suggestive linkage was later found to be a false-positive finding, this study supported the interest for chromosome 21 in AD research. At the same time APP was identified as the gene encoding the precursor of the Aβ peptides purified from the SPs in AD brains and it was localized to the linked chromosome 21 region[9,10].

The APP gene consists of 18 exons with the Aβ peptide encoded by parts of exons 16 and 17[9]. A heterogeneous group of proteins is transcribed from the gene as a result of both alternative splicing and a variety of post-translational modifications, including the addition of N- and O-linked sugars, sulfation, and phosphorylation[11,12,13,14,15]. Three major APP isoforms containing the Aβ sequence have been observed, i.e., the APP695, the APP751, and the APP770, of which the APP695 is the major brain isoform. At least five less abundant isoforms are generated by the alternative splicing of exons 7, 8, and 15.

APP is a ubiquitously expressed single transmembrane spanning protein with a large ectodomain and a small cytoplasmic tail with the Aβ peptide contained partly in the extracellular domain and partly in the transmembrane domain. The normal function of APP is unclear, but it is suggested that the APP protein is multifunctional with different roles in development and aging.

Two major proteolytic processing pathways exist to release Aβ from its precursor (Fig. 1). In the constitutive pathway, APP is cleaved by α-secretase within the Aβ domain between residues K687 and L688 (numbering according to the APP770 isoform)[16]. Through this pathway, no full-length Aβ is generated. In the amyloidogenic pathway, APP is cleaved by β-secretase between residues M671 and D672 and subsequently cleaved by γ-secretase either before I712 or before T714. The aspecific cleavage at the γ-secretase site leads to the generation of full-length Aβ peptides with 40 (Aβ40) or 42 (Aβ42) amino acids[17,18]. In many cells the most prominent pathway is the constitutive pathway. Neurons, however, have an intrinsic tendency to metabolize APP along the amyloidogenic pathways with Aβ40 as the predominant Aβ isoform produced[19,20,21]. As a general hypothesis it is believed that Aβ is a key player in the development of AD, and that all EOAD mutations are influencing the ratios or properties of the different Aβ isoforms in the brain[22].

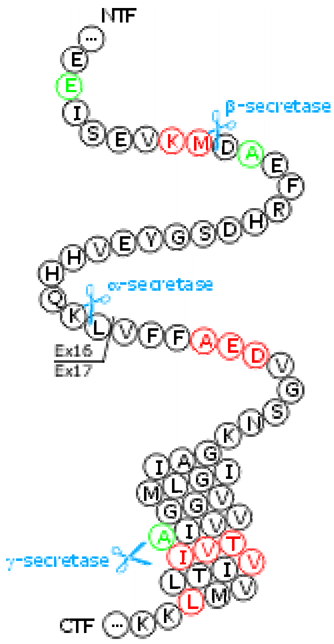

**FIGURE 1.** Schematic representation of exons 16 and 17 of APP. Amino acids with pathogenic missense mutations are shown in red and amino acids with not-pathogenic missense mutations are in green. For each mutation a hyperlink to the AD mutation database (http://molgen-www.uia.ac.be/ADMutations/) is included.

Independent of Aβ formation, both the constitutive and the amyloidogenic pathways result in the generation of C-terminal intracellular fragments during γ-secretase cleavage. These APP intracellular domains (AICDs) are highly labile fragments composed of 10 or 12 amino acids of the APP transmembrane domain and 47 amino acids of the cytoplasmic tail. It has been hypothesized that these AICDs may be transported to the nucleus where they play a role in physiological signalling through the transcriptional regulation of target genes. Using a reporter gene system it was shown that AICDs stabilized by the multidomain adaptor molecule Fe65 and bound to the histone acetyltransferase Tip60, were able to cause transcriptional activation[23,24]. Recently it was demonstrated that AICD in combination with c-Jun N-terminal kinase interacting protein-1 (JIP-1) also could activate gene expression in a Tip60-independent manner[25].

### Mutations in APP and Their Effect on the APP Processing

Mutations in APP are estimated to account for approximately 5% of all familial EOAD patients and are therefore a rare cause of AD. Mutation analysis of exons 16 and 17 of APP revealed the first APP mutation in 1991 in a family with patients suffering from hereditary recurrent cerebral hemorrhages with amyloidosis of the Dutch type (HCHWA-D)[26,27]. The identified G693Q mutation induced recurrent hemorrhagic strokes due to excessive Aβ deposition in the cerebral and leptomeningeal blood vessel walls[28]. No NFTs were observed in brains of HCHWA-D patients. The discovery of this mutation demonstrated that mutations in APP can lead to Aβ deposition and encouraged the mutation analysis of APP in EOAD families. Subsequently, several different APP missense mutations were identified in EOAD families[29]. To date, 16 different pathogenic mutations as well as 4 nonpathogenic polymorphisms have been described (Fig. 1). All APP mutations causing EOAD are located within exons 16 and 17 encoding the Aβ peptide. Moreover, all clear pathogenic mutations identified in APP are located at or near the three proteolytic cleavage sites involved in the processing of APP (Fig. 2). Mutations at these residues prevent the normal metabolism of APP and increase Aβ production by subtle different mechanisms. The Swedish double mutation APP670/671[30], immediately preceding the β-secretase cleavage site, induces increased cleavage by β-secretase to generate more Aβ40 and Aβ42. The different mutations at codons 714–717[29,31,32,33,34,35,36], occurring just C-terminal of the γ-secretase cleavage sites, appear in slightly different ways to enhance the production of Aβ peptides ending at residue 42. APP723, located more C-terminal, is predicted to alter the luminal transmembrane length and helical arrangement of the APP molecule and thus affect the γ-secretase cleavage site[37]. Three other mutations are located near the α-secretase site within the Aβ peptide[26,38,39]. In contrast to mutations near the β- or γ-secretase sites, these mutations do alter the amino acid sequence of the Aβ peptide. As a result these mutations are not causing classical AD phenotypes. The APP693 mutation described previously results in HCHWA-D, whereas the APP692 and APP694 mutations were identified in patients with cerebral hemorrhages due to congophilic amyloid angiopathy (CAA), and in EOAD patients belonging to the same family. *In vitro*, it was shown that the APP692 mutation generates more Aβ40 and Aβ42, presumably because the presence of the mutation hampers the α-secretase cleavage site[40]. Overall, all EOAD mutations in APP result in abnormal processing of APP leading to increased secretion of Aβ42. This Aβ42 peptide has been shown to aggregate more rapidly into fibrils[41], and was claimed to be the earliest and most abundant Aβ peptide in SPs[42,43,44].

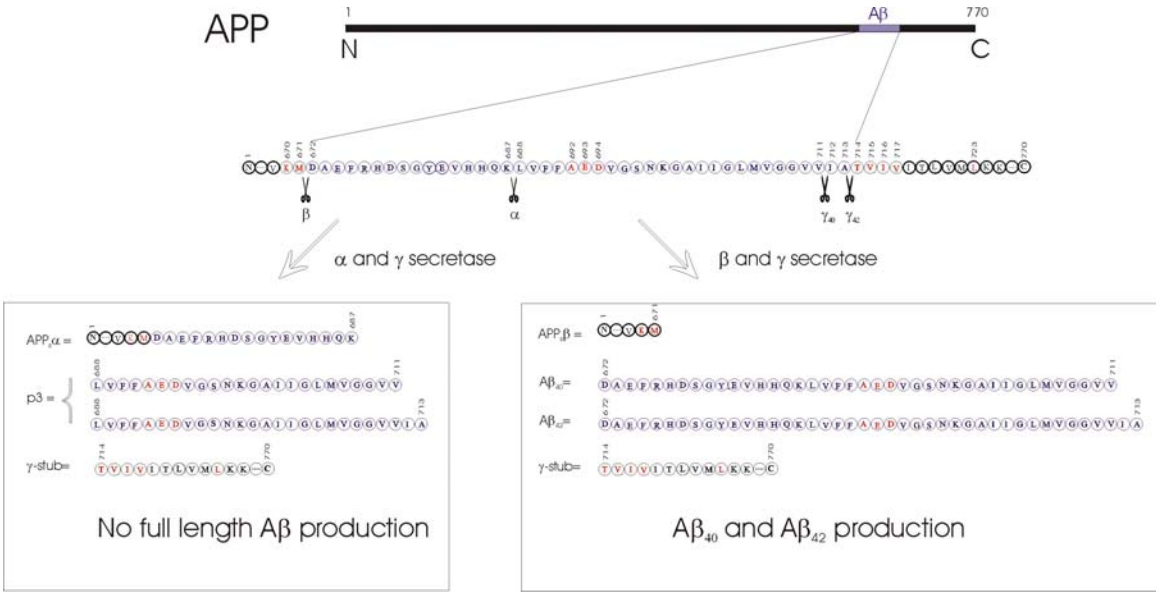

**FIGURE 2.** Proteolytic cleavage of APP770. The sequence within APP that contains Aβ as well as part of Aβ flanking sequence is expanded and shown by the single-letter amino acid code. Amino acids comprising the Aβ sequence are shown in blue, flanking amino acids in black. Amino acids with currently known missense mutations are indicated in red. The different proteolytic fragments resulting from protease activities of α, β, and γ–secretase are shown (APPs = soluble APP).

### PSENS

#### Identification of the PSENs: Gene and Protein Structure

Most EOAD families did not show linkage to chromosome 21 and mutations in APP were excluded[45,46]. This indicated that at least one other genetic locus for AD existed. As a result, genome-wide scans were initiated in several laboratories, leading to the identification of an AD locus on chromosome 14q24.3[47,48,49,50] and the identification of mutations in the PSEN1 gene[51]. Only a few months later, PSEN2 (located on chromosome 1q42.3) was identified based on its homology to PSEN1 and genetic linkage to this chromosomal region in a series of German AD-families originating from the Volga valley in Russia[52,53].

The PSEN1 and PSEN2 genes are very similar in structure, with ten exons (exons 3–12) comprising the coding sequence[51,54]. Less homology is found in the 5'UTR with three additional exons in PSEN1 and only two in PSEN2. For both genes several alternative transcripts were reported. In PSEN1 alternative use of the splice donor site for exon 3 results in the inclusion or exclusion of codons 26–29 (VRSQ)[55,56]. The inclusion of the VRSQ motif creates potential phosphorylation sites for protein kinase C and caseine kinase II. In PSEN2 the VSRQ motif is only partially conserved and not alternatively spliced[57]. In both PSENs exon skipping results in the in-frame deletion of exon 8, although for PSEN1 this has only been observed in leukocytes[51]. In PSEN2 truncated transcript lacking exon 3 and 4 were also identified[57]. Use of alternative start codons generates N-truncated proteins.

Like APP, PSEN proteins are ubiquitously expressed in all body tissues. The structure of the PSEN proteins is proposed to contain eight transmembrane (TM) domains with the N- and C-termini as well as the large hydrophilic loop linking TM6 and TM7 located cytoplasmic[58,59]. The overall amino acid identity of PSEN1 and PSEN2 is 67%, with the highest similarity found in the putative TM domains[51,53,60]. The PSEN holoproteins are proteolytically cleaved within exon 9 by a putative protease, designated presenilinase[61]. In PSEN1 this endoproteolytic cleavage generates N- and C-terminal heterogeneous fragments of 28 and 18 kDa, respectively, bound to each other to form a heterodimeric complex. The production of these endoproteolytic fragments seems to be tightly regulated, since they are produced in a 1:1 stoichiometry, and artificial overexpression of PSEN1 does not result in a linear overproduction of the smaller fragments. Cleavage of PSEN2 generates similar proteolytic fragments.

### Mutations in PSENs and Their Effect on the APP Processing

PSEN1 is the most important EOAD gene with 129 different mutations identified to date (http://molgen-www.uia.ac.be/ADMutations/) (Fig. 3). Based on the criteria to define autosomal dominant EOAD, estimated mutation frequencies of 18–50% have been reported[62,63,64]. Mutations in PSEN2 are a rare cause of EOAD (less than 1%) (Fig. 4)[63,64].

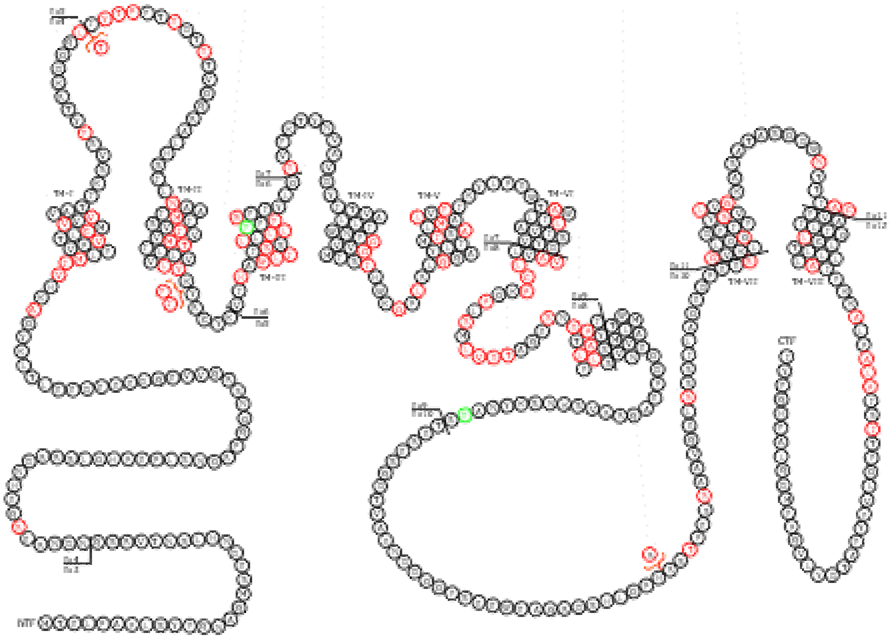

**FIGURE 3.** Schematic representation of PSEN1. Amino acids with pathogenic missense mutations are shown in red and amino acids with not-pathogenic missense mutations are in green. For each mutation a hyperlink to the AD mutation database (http://molgen-www.uia.ac.be/ADMutations/) is included.

Almost all PSEN mutations are missense mutations resulting in a single amino acid substitution. These missense mutations are scattered all over the protein, but their distribution is not random[22]: (1) most mutations occur in TM domains and in other residues that are well conserved between human PSENs and homologue PSENs of other species, (2) all mutations in TM2 line up on one side of the α-helix, suggesting that this helical face is important, probably in interacting with other TM domains or other TM proteins, and (3) a major cluster of mutations is present in exon 8 close to the endoproteolytic cleavage site.

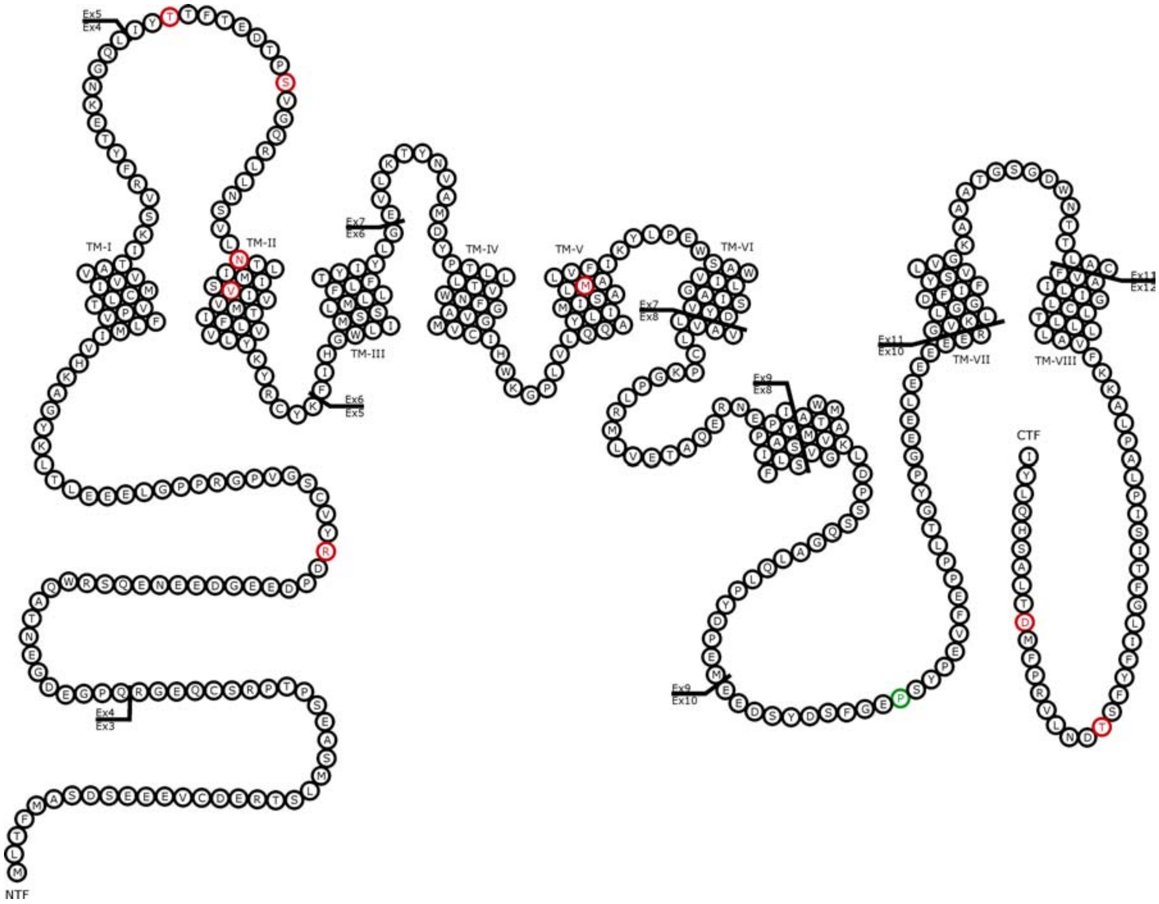

**FIGURE 4.** Schematic representation of PSEN2. Amino acids with pathogenic missense mutations are shown in red and amino acids with not-pathogenic missense mutations are in green. For each mutation a hyperlink to the AD mutation database (http://molgen-www.uia.ac.be/ADMutations/) is included.

In PSEN1, six exceptional mutations have been described: four deletions and two insertion mutations. The first group of mutations leads to the in-frame deletion of exon 9. The deletion of this exon was first reported as a result of a point mutation in the splice acceptor site of exon 10[65]. This mutation allows exon 8 to be fused in-frame to exon 10 thereby removing exon 9, and changing the amino acid at codon 290 (S290C), the splice-junction of exon 8 and exon 10. Later a 4.6-kb genomic deletion containing exon 9 was described in a Finnish pedigree leading to the same mutant protein[66]. Although this mutation abolishes the endoproteolytic processing of PSEN1 by presenilinase, it was shown that AD pathology is not caused by the aberrant processing of PSEN1, but results from the single amino acid change at the splice junction of exon 8 and 10[67]. The second deletion is a G deletion in the splice donor site of intron 4[68,69]. This mutation results in two out-frame deletion transcripts, lacking either the entire exon 4 or part of exon 4 due to cryptic splicing. In addition, an in-frame insertion transcript is produced by alternative splicing using a cryptic splice site in intron 4, leading to the insertion of a Thr codon between codons 113 and 114. Similar to the exon 9 deletions, the pathological function of this mutation is ascribed to the insertion of the single Thr codon, and not to the deletion transcripts that produce premature C-truncated proteins[69]. Recently, two other in-frame deletion mutations in PSEN1 have been identified. Sequence analysis of PSEN1 exons 4 and 6 revealed heterozygous deletions of ATCATG at codons 83 and 84 (deltaI83/M84)[70], and deletion of TAT at codon 167 (deltaI167)[71]. In 2001, two in-frame insertion mutations were identified in a referral-based series of AD[72]. An insertion of TTATAT in exon 6 is predicted to result in a missense substitution at codon 156 (Tyr156Phe) plus the insertion of an Ile and Tyr immediately after codon 156. The other insertion mutation results in the incorporation of an Arg codon at position 352 in exon 10. No out-frame mutations leading to alternative C-terminal sequences and premature stops were found.

In addition to these pathogenic missense mutations, two polymorphisms in the coding region of PSEN1 were reported[73,74]. The Glu318Gly substitution involves the last codon of exon 9 and is located in the middle part of the large hydrophilic loop of PSEN1. In a healthy Dutch elderly population, we estimated the allele frequency of this rare polymorphism at 1.6%. The other polymorphism, Phe175Ser in exon 6, was found in one patient from an Italian LOAD family, but this mutation did not segregate with the dementia in this family. In PSEN2 Pro334Arg was identified as a nonpathogenic missense mutation in addition to several silent polymorphisms[75].

All PSEN mutations, both single amino acid substitutions and small insertions and deletions, lead to aberrant γ-secretase cleavage of APP resulting in an overproduction of Aβ42. This was first noticed by an increase of Aβ42 in fibroblasts, in plasma and in brain of PSEN1 and PSEN2 mutation carriers[22]. Later, this finding was confirmed in medium from cells transfected with mutant PSEN. It was also shown that the expression of PSEN1 harboring alanine substitutions of two conserved aspartate residues at position 257 and 385 resulted in the inhibition of Aβ secretion and accumulation of APP C-terminal fragments[76]. From these studies it was hypothesized that PSEN1 may be an aspartyl protease required for the endoproteolysis of Aβ. Although it was long debated whether PSEN1 itself harbors γ-secretase function, or is an essential co-factor in the cleavage process, recent studies demonstrated that γ-secretase activity requires the formation of a stable high-molecular-weight protein complex containing three essential co-factors in addition to the endoproteolysed PSEN fragments. The first co-factor identified was nicastrin (NCSTN), a glycosylated integral membrane protein that is required for stabilization of the PSEN fragments[77]. Through genetic studies in *C. elegans*, two additional co-factors, anterior-pharynx defective protein 1 (APH-1) and presenilin enhancer 2 (PEN-2), were found[78,79]. It was shown that these four proteins are a minimal requirement for an effective cleavage of APP by γ-secretase, however other proteins or lipids could be rate-limiting for the overall activity of the complex in mammalian cells[80,81].

In general, increased production of Aβ42 by γ-secretase, resulting from mutations in both APP and PSEN, is in favor of the amyloid cascade hypothesis, stating that the production of more Aβ42 is a general phenomenon leading to AD pathology[22].

### APOE

#### Identification of APOE: Protein Isoforms and Their Risk for AD

The failure to detect mutations in APP, PSEN1, and PSEN2 in LOAD patients prompted many researchers to screen genetic markers at other loci in LOAD families. A positional cloning approach based on the comparison of affected individuals identified suggestive linkage to chromosome 19q13.2[82]. APOE, located close to the candidate region, was a strong candidate since immunoreactivity of APOE in AD brain lesions was previously shown[83], and APOE showed high affinity to Aβ *in vitro*[84].

The APOE gene consists of 4 exons encoding a glycoprotein of 299 amino acids. In the general population, 3 common sequence variations result in the production of 3 APOE isoforms. The most common variant, ε3, reflects the presence of a cysteine at codon 112, and arginine at codon 158 and is present in approximately 75% of Caucasians. A second coding variation, ε4, reflects the presence of an arginine at both codon 112 and 158, and is present in approximately 15% of Caucasians. In 10% Caucasians, a cysteine residue is present at both codons, resulting in variant ε2. Analysis of these isoforms in normal populations and in AD patients has consistently shown that the APOEε4 allele is associated with an increased risk for AD. First it was demonstrated to increase familial LOAD[84], but later the association was extended to sporadic LOAD[85] and familial and sporadic EOAD[86]. Several studies have shown that the risk associated with APOEε4 is dose dependant[87]. A meta-analysis showed a nearly 3 times increased risk for carriers with one APOEε4 allele, and a 15 times increased risk for homozygous APOEε4 carriers[88]. Moreover, the age at onset of individuals with two APOEε4 alleles is earlier than the onset of those without an APOEε4 allele[87]. Less consistent is the association of the APOEε2 allele with a protective effect against AD and a later disease onset. Although replicated in some studies, this finding was not always confirmed[89]. In total, the proportion of patients with dementia that is attributable to the APOEε4 allele was estimated to be 7–20%[90,91].

### Isoforms of APOE and Their Influence on the Properties of Aβ

In contrast to APP and the PSENs, APOE does not seem to influence the processing of APP, leading to more Aβ42 production. However, it is hypothesized that the different isoforms of APOE influence the properties of Aβ, since it was shown that APOE promotes Aβ fibril formation *in vitro*[92]. It was demonstrated that APOEε3 has a higher binding affinity for Aβ than has APOEε4[84,93], possibly leading to a better clearance of Aβ[93]. Also, subjects carrying one or more APOEε4 alleles have a higher Aβ plaque load than subjects with no APOEε4 alleles[94]. New insights into how APOE influences Aβ formation *in vivo* have come from studies using transgenic animals expressing mutations in the human APP gene that were bred onto an APOE knockout (-/-) background[95]. In the absence of APOE, there was less Aβ deposition and only diffuse plaques were observed. To investigate the effect of the different APOE isoforms on Aβ deposition, APP mutant mice were crossed with transgenic mice lacking endogeneous mouse APOE and expressing different human APOE isoforms in the brain[96]. The expression of human APOE in these mice suppressed the early deposition of Aβ. However, after 15 months, the amount of Aβ deposition, fibril formation, and the severity of neuritic pathology was clearly more profound in mice expressing human APOEε4 than in those expressing human the APOEε3 allele.

## GENOTYPE-PHENOTYPE CORRELATIONS IN FAMILIAL EOAD

A recent study compared age at onset and age at death in 90 individuals with dominantly inherited EOAD caused by PSEN1, PSEN2, and APP mutations[97]. Although similar mean disease duration of about 10 years was observed for all three genes, the mean age at onset and death were significantly earlier in PSEN1 and APP mutation carriers as compared to PSEN2 mutation carriers. Mean ages at onset were 44 ± 8 years in the PSEN1 group, 59 ± 7 in the PSEN2 group, and 49 ± 7 in APP mutation carriers. In families with APP717 or APP670/671 mutations, it was shown that the age at onset is modulated by APOE genotype, i.e., the APP mutation patients had earlier onset ages when they had inherited APOEε4 alleles[98,99]. No influence on the onset age was seen in families with the AD/CAA mutation APP692 and the HCHWA-D mutation APP693[100]. Also, no modification of onset age was observed for PSEN1 and PSEN2 mutation carriers[1,101].

In combination with the AD phenotype, additional neurological and neuropathologial symptoms are seen in some families with mutations in APP and the PSENs. These features include myoclonus, epileptic seizures, pyramidal and extrapyramidal signs, cerebellar signs, ataxia, and CAA[39,102,103,104,105,106]. One of the variant AD phenotypes is AD presenting with spastic paraparesis and large "cotton wool" plaques on neuropathological examination of the brain. This AD phenotype was first seen in a family with deletion of exon 9 of PSEN1 due to a 4.6-kb genomic deletion[66]. Patients with the deltaI83/M84 deletion mutation and the missense mutation P436Q also showed similar AD pathology[70,107]. It was suggested that all the spastic paraparesis-causing mutations have a larger effect on Aβ42 production than those mutations that lead to the more typical AD phenotype[108]. In another systematic neuropathological study on PSEN1-related EOAD, a strong correlation was shown between CAA and PSEN1 mutation codon position[109]. It was demonstrated that a severe degree of CAA was much more frequent among patients with a PSEN1 mutation occurring after codon 200.

These and several other hypotheses are proposed to explain aberrant phenotypes, mostly based on only few observations. With the increasing number of mutations in the known EOAD genes, more mutations will be identified that do not exactly fit with the previous data and hypothesis. As long as the underlying mechanism leading to the AD pathology is unclear, this will lead to rejection, or at least further adaptation of the existing theories.

### SUSCEPTIBILITY GENES FOR EOAD

In AD, as in several other complex diseases, association studies have become a popular way to study the effect of sequence variations in a candidate gene on the development of a disease. In these studies a statistical test is performed to detect whether a particular allele of a genetic marker is enriched in diseased individuals compared to unaffected controls. Over the last decade, more than 30 associations have been reported between AD and candidate genes on nearly every chromosome in the genome. However, few of the detected associations seem to be replicable when tested in independent samples, leading to increasing skepticism about the usefulness of genetic association analysis in general[110,111].

Recently, a relatively large-scale association study was performed with 60 markers in 54 genes, including 13 previously claimed to show AD association[112]. Only the APOEε4 allele, tested as a positive control, provided highly significant evidence for both allelic and genotypic association with AD. Although for other markers slightly positive results were obtained, the occurrence of false positive findings arising from the increasing number of statistical tests could not be ruled out. This study clearly illustrates the difficulties involved in association analysis as they are performed in several laboratories today. Studying the biological relevance of a genetic variation in a candidate gene will probably be indispensable to distinguish false positive findings from true risk factors for AD.

In this view, the genes responsible for autosomal dominant EOAD, i.e., APP, PSEN, and APOE are interesting candidate genes that could harbor variations in their intronic and regulatory regions, responsible for susceptibility to EOAD in the general population.

A genetic association between LOAD and a polymorphism in intron 8 of PSEN1 was reported with a twofold-increased risk for LOAD for the AA genotype[113]. This finding was replicated in some other LOAD populations[114,115,116], while others showed no association[117,118,119]. In a population-based series of EOAD, this intronic polymorphism as well as a functional promoter polymorphism located 22 bp upstream of the transcription initiation site P1 of PSEN1 (-22C/T) were analyzed for their contribution to EOAD[120]. The association with intron 8 was not replicated, but significant association was found with the –22C/T promoter variation. Luciferase reporter gene analysis demonstrated a neuron-specific 50% decrease in promoter activity for the –22C allele[121] and deletion mapping suggested that a 13bp region surrounding the –22C/T polymorphism harbors a binding site for a negative regulatory transcription factor[122]. Interestingly, the positive association of EOAD with PSEN1–22C/T was replicated in an independent EOAD sample[123]. Multiple studies suggested that genetic variability in the regulatory region of APOE could modulate the risk associated with the APOEε4 isoform. In population-based studies, genetic association with AD, independent of APOEε4, was reported for three APOE promoter variations (–491A/T, –427T/C, and –219T/G)[124,125,126]. Functional analysis showed that these polymorphisms alter the transcriptional activity of the APOE promoter due to differential binding of transcription factors. Although several studies attempted to confirm this association, most replication studies reported either absence of association, or association due to linkage disequilibrium with APOEε4[127,128,129,130,131,132,133,134].

In view of the amyloid cascade hypothesis, other relevant functional candidate genes for susceptibility to EOAD are the secretases involved in the APP processing or molecular partners of these proteases. Two years ago, a type I transmembrane aspartyl protease, named beta-site APP-cleaving enzyme (BACE), was identified as the long sought β-secretase involved in the cleaving of Aβ from APP[135,136,137]. Because the Swedish APP670/671 mutation close to the β-secretase cleaving site results in an increased production of Aβ[138], it was hypothesized that variations in BACE might contribute to the susceptibility of EOAD through a similar effect on the Aβ processing. However, association analysis performed by three independent groups failed to show significantly different allele or genotype distributions between EOAD patients and controls[139,140,141].

Like all pathogenic PSEN mutations, artificial missense mutations in a conserved hydrophilic domain in NCSTN strongly increased Aβ production whereas deletions in this domain inhibited Aβ production[77]. NCSTN is a type I transmembrane glycoprotein and was identified as a component of the PSEN-containing γ-secretase complex that is involved in the cleaving of APP (see section on PSEN)[77,142]. The contribution of genetic variation in NCSTN to EOAD was investigated using four polymorphisms capturing the four major NCSTN haplotypes. Genetic association analysis revealed that the risk of developing familial EOAD was modified by a significantly overrepresented NCSTN haplotype in familial EOAD patients (7%), in particular those patients lacking an APOEε4 allele (14%), compared to controls (3%). Because the four polymorphisms are either intronic or silent variations, no direct biological relevance for the association can be found. Perhaps a variation located in the regulatory region of NCSTN, influencing NCSTN expression levels, may be responsible for the observed association.

These examples of association studies with candidate genes that are functionally involved in fundamental processes in the development of AD, demonstrate the difficulties related to this approach. Either negative or conflicting results are found in different populations, or positive association results are obtained in a relatively small subgroup of the total sample, which makes it difficult to interpret the results. With the exception of the risk associated with the APOEε4 allele, none of the reported findings has been consistently replicated in all studies. Without denying the complex nature of a multifactorial disease, these results raise questions on the usefulness of this strategy in unraveling the genetic compounds of EOAD[143].

### TRANSGENIC MOUSE MODELS FOR EOAD

The SPs and NFTs found in postmortem AD brains represent the end-stage of the AD disease process. The study of human AD patients will therefore be insufficient to obtain clear insights into the early pathogenic events that are leading to AD pathology. Attempts to solve this problem have focused on the development of transgenic EOAD mice. These animal models offer an excellent opportunity to study the underlying mechanism causing brain dysfunction in AD and they also provide an important tool to identify new therapeutic targets and novel treatments. During the past decade, a number of different transgenic mice models that (over)express one or more mutant human proteins (APP, PSEN, MAPT) associated with familial AD have been created. Several of these mice show cognitive deficits and increased Aβ production, but at present there is no single transgenic mouse exhibiting the combination of all characteristic AD neuropathological traits, such as overt neuronal loss, SPs, and extensive NFT pathology.

The first attempts to create EOAD transgenic mice focused on transgenic APP mice, because different APP mutations had shown to alter Aβ processing *in vitro*. Transgenic mice with wild-type[144] APP and APP with different clinical mutations[145,146], as well as mice with intracellular Aβ peptide[147] or the C-terminal domain of APP[148], were generated. Most extensive Aβ deposition was seen in the APP V717F mice[146] and, although less pronounced, in the Swedish APP K670N/M671L mice[145]. Measurements of the different APP metabolites in these mice brains, demonstrated that increased Aβ42 levels correlated with the formation of SPs.

Because of the significant differences between human and rodent cognition, it is difficult to evaluate the effect of Aβ deposition on the development of AD in these mice brains. Many of the diagnostic features of AD involve loss of functions that are uniquely human, such as language disturbances or loss of reasoning ability. Therefore, most behavioral studies with transgenic mice have focused on learning and memory, as well as some general measurement of neurological health. Deficits in spatial memory were reported for nearly all transgenic APP mice, but none of the APP transgenic mice reported gross impairment on neurological functioning[149].

To establish that the PSEN mutations also alter APP processing, transgenic mice overexpressing wild type and mutant PSEN were constructed[150,151,152]. Analysis of these mice showed that overexpressing mutant, but not wild type PSEN1, selectively increased brain Aβ42. Moreover, accelerated Aβ deposition was seen in the brains of transgenic mice co-expressing mutant PSEN1 A246E and APP K670N/M671L[153]. These results were in favor of the hypothesis that all mutations in EOAD genes are causing AD by altering the normal processing of APP.

Knockout mice provided even more insight into the function of wild type and mutant EOAD proteins. Deletion of the APP gene in mice resulted in neither early mortality, nor appreciable morbidity, although these mice exhibited reactive gliosis and changes in locomotor behavior[154]. The lack of vital consequence of APP deletion *in vivo* may result from the fact that mammals express proteins closely homologous to APP, the APP-like proteins (APLPs)[155]. In contrast to APP null mice, PSEN1 deficient mice are not viable[156]. Because embryonic lethality precludes further analysis of PSEN1 on APP metabolism *in vivo*, brain cultures were derived from PSEN1 null-embryo's[157]. *In vitro*, cleavage of the extracellular domain of APP by α- and β-secretase was not affected by the absence of PSEN1, but the activity of γ-secretase was prevented. This inhibition caused an accumulation of C-terminal fragments of APP and a dramatical decrease in Aβ production. In 2001, PSEN1 conditional knockout mice were created and raised to adulthood[158]. In these animals the levels of C-terminal fragments of APP were 30 times increased as compared to normal, and Aβ40 and Aβ42 peptides were significantly reduced. These *in vitro* and *in vivo* data confirmed the hypothesis that PSEN1 facilitates the γ-secretase cleavage of APP.

PSEN2 knockout mice are viable and phenotypically normal, and the absence of PSEN2 did not affect the Aβ production in neuronal cultures[159,160].

To model the formation of NFT pathology, and to investigate the relationship between Aβ deposition and NFT formation, transgenic mice with wild type and mutant human MAPT were created. In human, the MAPT gene is alternatively spliced to generate six different transcripts. Inclusion or exclusion of exon 10 generates a tau protein with three or four microtubule binding repeats (3R or 4R). Adult human brain maintains approximately equal amounts of 4R and 3R tau, whereas in adult mice only 4R tau is present[161]. Two important experiments showed a relationship between the formation of NFT and Aβ deposition. When Aβ was given by injection to the CA1 region of the hippocampus of transgenic MAPT P301L mice, NFT developed in the respective cell bodies of projection neurons in the amygdala[162]. In addition, transgenic mice expressing the MAPT P301L mutation were crossed with transgenic APP K670N/M671L mice. Compared to transgenic mice expressing only mutant tau, these double transgenic mice showed enhanced formation of NFT in the limbic system and the olfactory cortex[163]. These experiments suggest that either APP or Aβ influences the formation of NFT.

### CONCLUSION

More than 10 years ago, the first mutations responsible for EOAD were described. Since then, it became clear that not only AD in general, but even autosomal dominant EOAD, is a complex genetic disorder characterized by allelic and locus heterogeneity. The four well-established genetic factors involved in EOAD are the APP gene, the two PSEN genes, and the ε4 allele of the APOE gene. All these genes were identified using positional cloning strategies in families segregating AD. Association studies with candidate genes have been less successful, and could only confirm the positive association of APOEε4. Recent estimates suggest that the four established AD genes account for less than 30% of the genetic variance in age at onset for AD, and predict that numerous additional AD genes may exist[164]. A classical approach, performing linkage analysis in sufficiently large family samples, is still the most promising approach to identify these novel AD genes. In EOAD, several families have been reported in which mutations in the known AD genes were excluded[63,165], but no new chromosomal loci have been identified to date. More progress is made in LOAD, where several genome scans have revealed candidate loci on chromosome 9, 10, and 12[166,167,168,169,170,171,172,173,174]. Although a number of genes from these chromosomal loci are proposed as potential AD genes, i.e., insuline degrading enzyme (IDE), α2 macroglobuline (A2M), and more recently urokinase-type plasminogen activator (uPA), none of the candidate genes have shown indisputable results in mutation and association studies[175,176]. Additional genome scans, in both EOAD and LOAD families, will therefore be needed to identify and further refine these AD-loci and identify novel AD-genes. It is expected that the identification of these novel genes will enhance our understanding in AD pathology and contribute to the development of novel therapeutic strategies for this devastating disease.

### ACKNOWLEDGMENT

The research performed by the Neurogenetics group of the Department of Molecular Genetics VIB8 was funded by the Special Research Fund of the University of Antwerp (UA-BOF), the Fund for Scientific Research Flanders (FWO-F), the InterUniversity Attraction Poles (IUAP) program P5/19 of the Federal Office of Scientific, Technical and Cultural Affairs (OSTC), the International Alzheimer Research Foundation (IARF) and The Medical Foundation Queen Elisabeth, Belgium; the Netherlands Organization for Scientific Research (NWO), The Netherlands; and the Alzheimer Association USA (Grant # IIRG-00-2190). R.R. is a PhD fellow and M.C. a postdoctoral fellow of the FWO-F.
